# Smoking Cessation Smartphone App Use Over Time: Predicting 12-Month Cessation Outcomes in a 2-Arm Randomized Trial

**DOI:** 10.2196/39208

**Published:** 2022-08-18

**Authors:** Jonathan B Bricker, Kristin E Mull, Margarita Santiago-Torres, Zhen Miao, Olga Perski, Chongzhi Di

**Affiliations:** 1 Division of Public Health Sciences Fred Hutch Cancer Center Seattle, WA United States; 2 Department of Psychology University of Washington Seattle, WA United States; 3 Department of Statistics University of Washington Seattle, WA United States; 4 Department of Behavioural Science and Health University College London London United Kingdom

**Keywords:** acceptance and commitment therapy, ACT, digital interventions, eHealth, engagement, iCanQuit, QuitGuide, mobile health, mHealth, smartphone apps, trajectories, tobacco, smoking, mobile phone

## Abstract

**Background:**

Little is known about how individuals engage over time with smartphone app interventions and whether this engagement predicts health outcomes.

**Objective:**

In the context of a randomized trial comparing 2 smartphone apps for smoking cessation, this study aimed to determine distinct groups of smartphone app log-in trajectories over a 6-month period, their association with smoking cessation outcomes at 12 months, and baseline user characteristics that predict data-driven trajectory group membership.

**Methods:**

Functional clustering of 182 consecutive days of smoothed log-in data from both arms of a large (N=2415) randomized trial of 2 smartphone apps for smoking cessation (iCanQuit and QuitGuide) was used to identify distinct trajectory groups. Logistic regression was used to determine the association of group membership with the primary outcome of 30-day point prevalence of smoking abstinence at 12 months. Finally, the baseline characteristics associated with group membership were examined using logistic and multinomial logistic regression. The analyses were conducted separately for each app.

**Results:**

For iCanQuit, participants were clustered into 3 groups: “1-week users” (610/1069, 57.06%), “4-week users” (303/1069, 28.34%), and “26-week users” (156/1069, 14.59%). For smoking cessation rates at the 12-month follow-up, compared with 1-week users, 4-week users had 50% higher odds of cessation (30% vs 23%; odds ratio [OR] 1.50, 95% CI 1.05-2.14; *P*=.03), whereas 26-week users had 397% higher odds (56% vs 23%; OR 4.97, 95% CI 3.31-7.52; *P*<.001). For QuitGuide, participants were clustered into 2 groups: “1-week users” (695/1064, 65.32%) and “3-week users” (369/1064, 34.68%). The difference in the odds of being abstinent at 12 months for 3-week users versus 1-week users was minimal (23% vs 21%; OR 1.16, 95% CI 0.84-1.62; *P*=.37). Different baseline characteristics predicted the trajectory group membership for each app.

**Conclusions:**

Patterns of 1-, 3-, and 4-week smartphone app use for smoking cessation may be common in how people engage in digital health interventions. There were significantly higher odds of quitting smoking among 4-week users and especially among 26-week users of the iCanQuit app. To improve study outcomes, strategies for detecting users who disengage early from these interventions (1-week users) and proactively offering them a more intensive intervention could be fruitful.

## Introduction

### Background

User engagement in digital behavior change interventions has predicted improved treatment outcomes across a wide variety of domains, including mental health, physical activity, dietary change, weight loss, alcohol use, and smoking cessation [1-9]. A central challenge in creating effective digital health behavior change interventions is that a large proportion of users disengage early from these interventions, thereby contributing to low treatment success rates [2,10,11]. Given the importance of user engagement, designing strategies to increase engagement has been a priority of digital behavioral health interventions [2,7,8,12,13].

Within the domain of cigarette smoking, smartphone apps for smoking cessation have become a ubiquitous intervention approach for which user engagement can be readily measured. Nearly 500 English language smartphone apps for smoking cessation have been downloaded more than 33 million times since 2012 (R Nelson, SensorTower.com, personal communication, April 15, 2020). Higher user engagement in smartphone interventions for smoking cessation is predictive of cessation outcomes [11,14-16]. Although this positive association may partly be driven by self-selection bias (or reverse causation), as users have not been randomized to different levels of engagement, a certain level of exposure to the intervention’s active ingredients is logically necessary for successful behavior change [2]. This logic has led to a body of work focusing on identifying design strategies that promote user engagement. For example, tailoring content to individuals’ characteristics or unique situations, interactivity (eg, through conversational agents) [17], and credibility have been found across a range of studies using mixed methods to be important for engagement with smoking cessation interventions.

Although smartphone intervention engagement is usually measured by the number of log-ins, little is known about how users engage with smartphone interventions *over time* and whether those temporal patterns predict higher odds of smoking cessation. In the educational literature, a well-documented finding is that learning new material becomes more effective when it occurs over a longer period [18]. This process, called spaced practice, increases the variability in learning and remembering new information [19]. The purpose of this study was to determine, in the domain of smartphone apps for smoking cessation, whether user engagement over time leads to improved cessation outcomes.

Smartphone apps for smoking cessation are commonly available for participants to use at will, resulting in high variations in use trajectories over time. For example, some users may follow a trajectory of logging in several times within the first few days of starting an intervention and then never return. Others may follow a trajectory in which they log in consistently and then gradually taper off. Other users may follow a trajectory in which they consistently log in over the course of several months. Conceivably, some groups of individuals might follow unique use trajectories over time that are associated with differential health outcomes. For example, people who log in consistently over the course of many months might have higher cessation rates, because they have consistently benefited from the information and skills presented in the app.

There is a dearth of studies analyzing use trajectories for digital smoking cessation interventions. We are aware of 3 publications for SMS text messaging interventions [20-22], 4 publications for website interventions [5,23-25], and none for smartphone interventions. Regarding SMS text messaging interventions, a study identified 5 distinct use trajectories of an SMS text messaging–based smoking cessation program over the 6 weeks after quit date, namely high engagement, increasing engagement, rapid decrease, delayed decrease, and low engagement [20]. The study found that the high engagement and increasing engagement groups were more likely than the other groups to quit smoking over the course of 6 weeks. Within the context of smoking cessation websites, our group conducted a functional clustering analysis of log-in data from both arms of a large (N=2637) randomized trial of 2 website interventions for smoking cessation (WebQuit and Smokefree), with a primary outcome of 30-day point prevalence smoking abstinence at 12 months [24]. Compared with 1-week WebQuit users, 5- and 52-week users had 57% higher odds (odds ratio [OR] 1.57, 95% CI 1.13-2.17; *P*=.007) and 124% higher odds (OR 2.24, 95% CI 1.45-3.43; *P*<.001), respectively, of being abstinent at 12 months. The 5-week use of either website predicted higher odds of quitting smoking, with the highest odds for 52-week WebQuit users. These results suggest that experimental testing strategies to increase digital intervention engagement for 4 more weeks (ie, from 1 week to 5 weeks) would be valuable. Studying distinct groups of use trajectories can help identify which use patterns are beneficial and thereby make recommendations for future program use. These results will help inform the design of more engaging digital interventions for smoking cessation, with the ultimate goal of a higher likelihood of cessation.

If we can identify smartphone intervention use trajectories that predict cessation, understanding the sociodemographic characteristics of individuals who tend to follow more or less successful trajectories is important. Knowing the characteristics of individuals who are likely to have certain engagement patterns might allow researchers and intervention designers to tailor smartphone interventions according to users’ unique baseline characteristics. There is an emerging literature of randomized trial designs that algorithmically use baseline characteristics predictive of treatment outcomes in the design of tailored interventions [26,27]. Although studies have found that being female, being older, and having a higher education are generally consistent predictors of greater digital intervention use [28-31], very little is known about the user characteristics that are associated with different patterns of use over time [32,33]. For example, a study found that being female and having higher baseline motivation were associated with more consistent log-in trajectories [34]. For the WebQuit website intervention, we found that smoking for at least the past 10 years and screening negative for anxiety predicted a 90% higher odds (OR 1.90, 95% CI 1.14-3.14) and a 56% higher odds (OR 1.56, 95% CI 1.06-2.33), respectively, of being a 52-week user (compared with being a 1-week user) [24]. Regarding smoking cessation smartphone apps, we are aware of no literature on baseline predictors of their use patterns over time.

We recently developed and tested iCanQuit, a smartphone app for smoking cessation based on acceptance and commitment therapy (ACT), a behavioral approach that teaches skills for allowing cravings to smoke to pass without smoking, which is conceptually distinct from the US Clinical Practice Guidelines (USCPG)–based approaches that teach avoidance of urges [35]. In a large 2-arm randomized trial, iCanQuit was compared with QuitGuide, a USCPG-based smartphone app. At the 12-month follow-up, iCanQuit was 1.5 times more efficacious than QuitGuide for smoking cessation among 2415 adults who smoked (36% racial or ethnic minority groups) from all 50 US states [35]. The iCanQuit study was the first full-scale randomized controlled trial with long-term follow-up to show that a smartphone app was efficacious for smoking cessation.

### Objectives

Using data from the iCanQuit parent randomized trial, this study identified the following: (1) distinct groups of smartphone app log-in trajectories, (2) their association with the 12-month smoking cessation outcome, and (3) baseline sociodemographic user characteristics that are associated with different use trajectory groups. Log-in trajectories (ie, log-ins over time) are a generalizable metric that can be useful for other digital intervention researchers—agnostic of the intervention-specific content contained in any one app. The overall goal of this study is to inform the design of future smartphone health interventions that could be more efficacious by identifying trajectory groups in need of further intervention. To accomplish these aims, in this study we analyzed 182 consecutive days of log-in data from both arms of the large (N=2415), 2-arm randomized trial of iCanQuit versus QuitGuide smartphone app interventions for smoking cessation (NCT02724462).

## Methods

### Design

A total of 2415 individuals were enrolled in the 2-arm iCanQuit randomized controlled trial for smoking cessation, with full protocol details previously described [12]. In brief, a racially and ethnically diverse sample of 2415 adult daily smokers from all 50 US states was randomized 1:1 to receive access to an ACT-based smartphone app (iCanQuit) or a USCPG-based smartphone app (QuitGuide) for smoking cessation. Data for this analysis were from the 2133 individuals who logged into their assigned app at least once and had a complete 182 days of engagement data available.

### Eligibility Criteria

Eligibility criteria included individuals who (1) were aged ≥18 years; (2) smoked 5 or more cigarettes per day for the past year; (3) wanted to quit smoking within the next 30 days; (4) if concurrently using any other tobacco products, wanted to quit all tobacco products within 30 days; (5) were interested in learning skills to quit smoking and willing to be randomized to either treatment condition; (6) had daily access to their own smartphone; (7) knew how to download smartphone apps; (8) were willing and able to read in English; (9) had never used QuitGuide and not currently using another smoking cessation treatment; (10) had never participated in our prior studies; (11) had no household members already enrolled; (12) were willing to complete outcome surveys, and (13) could provide contact information for themselves and 2 relatives.

### Recruitment, Enrollment, and Follow-up

Adults were recruited nationally via Facebook advertisements, a survey sampling company, search engine results, and friend or family referrals. Participants completed an encrypted web-based screening survey and were notified of their eligibility via email. They then clicked on their secured emailed link to the study website where they provided consent and completed the baseline survey. At each enrollment step, the study was presented as a comparison of 2 smartphone apps for smoking cessation.

Participants were randomized (1:1) to iCanQuit or QuitGuide using randomly permuted blocks of sizes of 2, 4, and 6, stratified by smoking frequency (≤20 vs ≥21 cigarettes per day), education (≤high school vs ≥some college), race or ethnicity (minority race or ethnicity vs non-Hispanic White), and depression screening (Center for Epidemiologic Studies Depression score ≤15 vs ≥16) [31]. Random assignments were concealed from participants throughout the trial. The random allocation sequence was generated by a database manager and implemented automatically by the study website. Neither research staff nor study participants had access to the upcoming randomized study arm assignments. In both arms, participants could access their interventions from the moment of randomization and beyond (ie, after the end of the 12-month follow-up period). All participants provided their consent on the web and were compensated with up to US $105 for completing study data collection. The data retention rate was 88% (1886/2133) and differed slightly between arms (90% in QuitGuide vs 87% in iCanQuit; *P*=.01).

### Interventions

#### iCanQuit

Participants randomized to the iCanQuit arm received access to download the iCanQuit smartphone app (version 1.2.1). iCanQuit intervenes on the ACT-focused processes of acceptance of internal cues to smoke and enacting one’s values that guide smoking cessation [12]. The acceptance component of the app teaches skills to accept physical sensations, emotions, and thoughts that trigger smoking by distancing oneself from thoughts about smoking (“cognitive defusion”), mindfulness skills, and flexible perspective taking. The values component of the app teaches skills for determining the core life domains that motivate quitting smoking (eg, family, health, and spirituality) and taking repeated small actions within these domains (eg, playing with grandchildren) to develop a smoke-free life. The program is self-paced, and the content is sequentially unlocked across 8 levels. Each of the first 4 levels is made accessible immediately after the prior level is completed, whereas each of the last 4 levels is only unlocked upon recording 7 consecutive days without smoking. If a participant lapses, the program encourages (but does not require) them to set a new quit date and return to the first 4 levels for preparation. The program also includes on-demand tools to help in coping with smoking urges, tracking the daily number of cigarettes smoked, and urges passed without smoking. Content was presented in a sequenced format with short paragraphs of text and some audio or visual content for experiencing ACT concepts.

#### QuitGuide

Participants randomized to the QuitGuide arm received access to download the QuitGuide smartphone app (version 1.2.2). QuitGuide content is delivered in four main sections: (1) “Thinking about quitting,” which focuses on motivations to quit by using reason and logic such as identifying reasons to quit and providing information on the health consequences of smoking and quitting; (2) “Preparing to Quit,” which helps users develop a quit plan, identify smoking behaviors, triggers, and reasons for being smoke free, and social support for quitting; (3) “Quitting,” which teaches skills for avoiding cravings to smoke; and (4) “Staying Quit,” which presents tips, motivations, and actions to stay smoke free and skills for coping with slips. No smoking cessation medications or coaching was provided in either intervention arm [12]. Content was presented in a sequenced format with short paragraphs of text.

### Study Measures

#### Baseline Characteristics and Covariates

Data collected at baseline included age, gender, race, ethnicity, education, employment, income, marital status, and sexual orientation. Study participants completed validated positive screening tools to assess mental health, including depression [31], panic [32], and posttraumatic stress disorder [33]. Alcohol consumption and heavy drinking were assessed using the Quick Drinking Screen [34]. Smoking behavior variables included nicotine dependence (measured using the Fagerström Test for Nicotine Dependence) [35], number of cigarettes smoked per day, years of smoking, use of e-cigarettes, quit attempts, and relationships with other smokers. Acceptance of internal cues to smoke was measured via the Avoidance and Inflexibility Scale (adapted from the study by Gifford et al [36]), using means of the three 9-item subscales that assess one’s willingness to experience physical sensations, emotions, and thoughts that cue smoking. The items are rated on a 5-point scale from 1=“not at all” to 5=“very willing” and averaged, with higher scores indicating greater acceptance. A sample physical sensation item was “How willing are you to notice these bodily sensations without smoking?” and items from the emotions and thoughts subscales were similar, substituting “feelings” or “thoughts” for “bodily sensations.” Valued living was measured using the 10-item Valuing Questionnaire [37], designed to assess the extent of personal values enactment. Each item is rated on a 7-point scale ranging from 0=“not at all true” to 6=“completely true.” Scores were averaged, and 2 distinct factors were derived: progress and obstruction, with higher scores indicating either greater progress or greater obstruction toward valued living, respectively. A sample progress item was “I worked toward my goals even if I didn’t feel motivated to” and a sample obstruction item was “I was basically on auto-pilot most of the time.”

#### Engagement: Baseline to Day 182 Log-ins

Engagement with the assigned app was measured objectively using Google Analytics. The measure of engagement was the number of days each application was opened, which was consistent with other digital interventions’ measures of engagement [7,24,25]. For each participant, time- and date-stamped log file records of each page opening were recorded. For this analysis, we used a binary measure indicating whether each participant logged in at least once each day (ie, had at least one log-in recorded in the log file data). Using this method, each participant had a 0/1 code for each day for 182 days from the date of randomization. Owing to a technical error in the Google Analytics system, only the first 182 days of engagement data were available for the study sample.

#### Smoking Cessation Outcome: 12 Months

The parent trial’s primary smoking cessation outcome was specified a priori as self-reported complete case 30-day point prevalence abstinence (PPA) at the 12-month follow-up. As reported in the parent trial, for the primary outcome of 30-day PPA at the 12-month follow-up, iCanQuit participants had 1.49 times higher odds of quitting smoking as compared with QuitGuide participants (293/1040, 28.17% abstinent vs 225/1067, 21.08% abstinent; OR 1.49, 95% CI 1.22-1.83; *P*<.001). Note that when missing data were coded as smokers, 12-month 30-day PPA results were very similar (293/1214, 24.13% abstinent for iCanQuit vs 225/1201, 18.73% abstinent for QuitGuide; OR 1.40, 95% CI 1.14-1.71; *P*<.001).

### Statistical Analyses

The analyses were conducted separately for each app. As mentioned in the engagement measurement, log-in data were summarized as a binary time series indicating log-in occurrence on each day of the first 6 months (ie, 182 days) of using the application, from the date of randomization of each participant. Next, log-in time series were presmoothed as the average number of days logged in over a window of 7 previous days [24,36]. This type of dense trajectories data is known as functional data. We applied functional clustering based on functional principal component (FPC) analysis. Specifically, we conducted an FPC analysis [37] by smoothed covariance to summarize each participant’s log-in trajectory using a set of low-dimensional FPC scores. We chose to retain the first 3 and 4 FPC scores for clustering in the iCanQuit and QuitGuide arms, respectively, based on a minimum threshold of 90% for the percentage of variance explained. Trajectories were clustered using the Clustering for Large Applications algorithm [38] into *k*=2 and 3 groups in each arm, which met a minimum prediction strength [39] threshold of 0.6. The Clustering for Large Applications procedure does not rely on parametric assumptions on the shapes of trajectories and is capable of handling densely recorded longitudinal data and complex missing data patterns. We then examined the cluster solutions in each arm for a minimum group size ≥5% of the sample and reasonable separation of the mean log-in trajectories among groups to determine the optimal number of groups for each.

After determining distinct trajectory groups, smoking cessation rates were compared among the groups using logistic regression, with the lowest use group as the reference group. Baseline characteristics with significant univariate associations with cessation were considered as covariates, to control for characteristics that may confound the association between trajectory group and cessation [40]. Thus, the aim of the analysis, as guided by the study’s scientific questions, was to understand the unique prediction of the 12-month cessation outcome by trajectory group membership. A shared set of covariates (ie, all baseline characteristics) was considered for each treatment arm. We conducted stepwise logistic regression using Akaike Information Criterion to determine the subset of covariates to be included in the final adjusted model [41]. Finally, baseline characteristics were compared among the groups. Those with significant univariate association with trajectory group membership were considered in stepwise selection, using Akaike Information Criterion, of an adjusted multinomial logistic regression model (iCanQuit arm) or logistic regression model (QuitGuide arm) to determine the best baseline predictors of group membership. All statistical tests were 2-sided, with α=.05, and analyses were conducted in R (version 4.0.3; R Foundation for Statistical Computing [42]), using the R packages “refund” [43] for FPC analysis, “fpc” [44] for prediction strength, and “nnet” [41] for multinomial logistic regression.

### Ethics Approval

All study activities were approved by the institutional review board at the Fred Hutchinson Cancer Research Center (approval number IR-8317).

### Description of Sample

Multimedia Appendix 1 shows the baseline demographics and participant characteristics in both the iCanQuit and QuitGuide arms. The mean (SD) age at enrollment was 37.8 (10.8) years. Furthermore, 70.28% (1499/2133) of the participants were female and 35.72% (762/2133) of the participants reported racial and ethnic minority backgrounds. There were 40.46% (863/2133) of the participants with a high school or less education. Regarding smoking, 74.54% (1590/2133) of the participants smoked more than half a pack (at least 11 cigarettes) per day. Less than half (785/2030, 38.67%) of the participants had made a quit attempt in the last year, and 82.47% (1759/2133) of the sample had been smoking for >10 years, with an average Fagerström Test for Nicotine Dependence score of 5.86 (moderate nicotine dependence; SD 2.04). There were no statistically significant differences between the 2 arms for any baseline variable (all *P*>.05).

### Description of Distinct Groups of Trajectories

**Figure 1:**
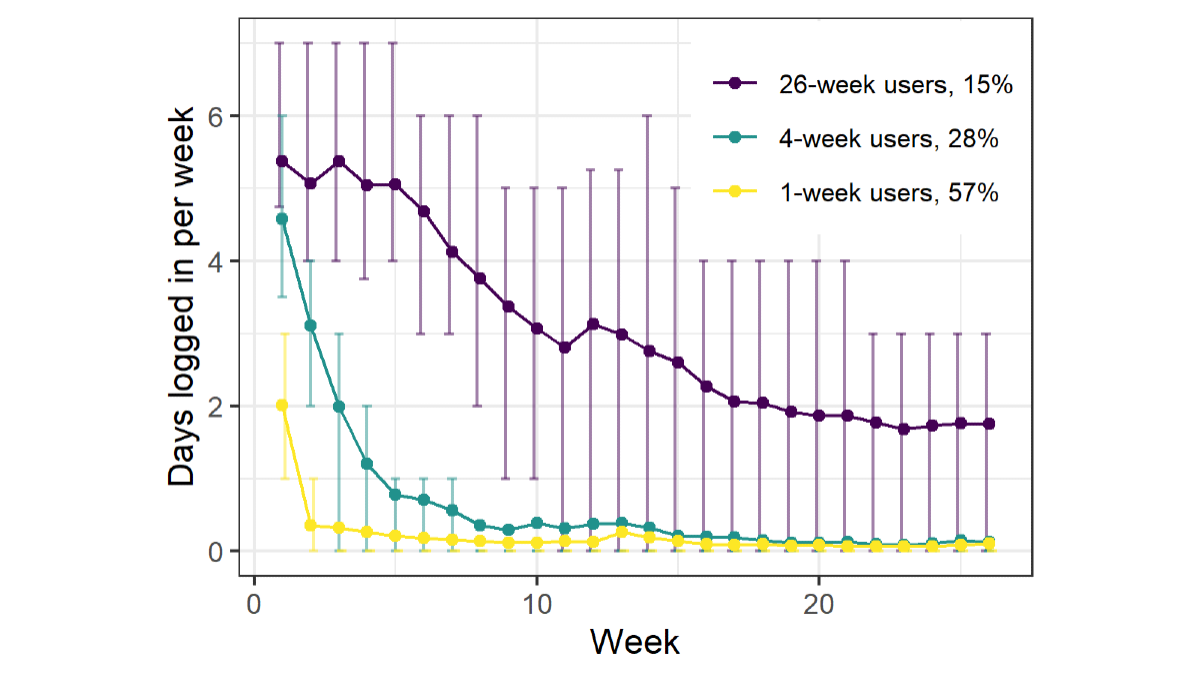
Mean weekly log-ins for each trajectory group from the iCanQuit arm. Error bars represent IQRs.

**Figure 2:**
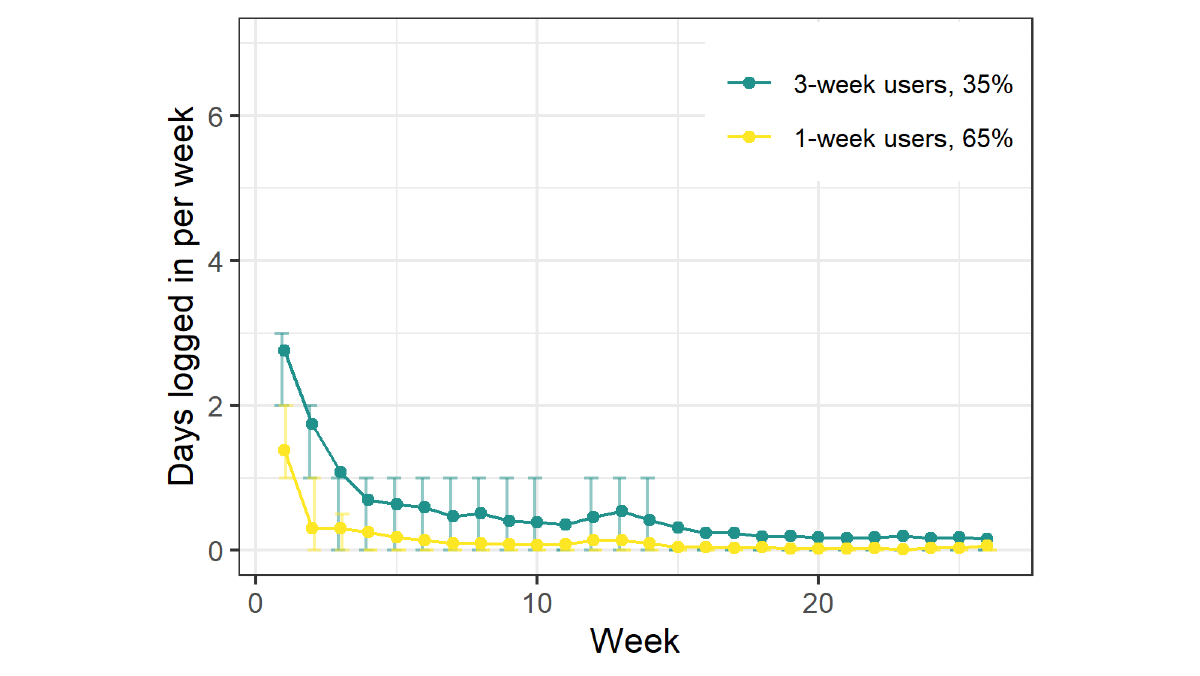
Mean weakly log-ins for each trajectory group from the QuitGuide arm. Error bars indicate IQRs.

The functional clustering analysis of 26 weeks of log-ins revealed 3 distinct groups of trajectories for iCanQuit versus 2 distinct groups for QuitGuide. Log-in patterns are shown in Figure 1 (for iCanQuit) and Figure 2 (for QuitGuide). For iCanQuit (Figure 1), the first trajectory group (610/1069, 57.06%) logged in a mean of 2.0 days in the first week and then had <1 mean log-in day in weeks 2 and beyond. They were termed “1-week users.” The second trajectory group (303/1069, 28.34%) logged in a mean of 4.6 days in week 1, a mean of 3.1 days in week 2, a mean of 2.0 days in week 3, a mean of 1.2 days in week 4, and then had <1 mean log-in day in weeks 5 and beyond. They were termed “4-week users.” The third trajectory group (156/1069, 14.59%) logged in a mean of 5.0 to 5.4 days per week in weeks 1 through 5, a mean of 3.1 to 4.7 days per week in weeks 6 through 10, tapering to a mean of twice every week starting week 17, and continuing in this pattern until week 26. They were termed “26-week users.”

For QuitGuide (Figure 2), the first trajectory group (695/1064, 65.32%) logged in a mean of 1.4 days in the first week and then had <1 mean log-in day in weeks 2 and beyond. As with iCanQuit, they were termed “1-week users.” The second trajectory group (369/1064, 34.68%) logged in a mean of 2.8 times in week 1, a mean of 1.7 times in week 2, a mean of 1.1 times in week 3, and then had few log-ins after that. They were termed “3-week users.”

### Trajectory Membership Prediction of Smoking Cessation Outcome

Table 1 shows each intervention arm’s trajectory group membership as a predictor of 30-day PPA at the 12-month follow-up, after controlling for all baseline covariates included in the statistical model. For iCanQuit, abstinence rates for the 3 trajectory groups were 23% for 1-week users, 30% for 4-week users, and 56% for 26-week users. Compared with the 1-week users, the 4-week users had 50% higher odds (OR 1.50, 95% CI 1.05-2.14; *P*=.03), whereas 26-week users had 397% higher odds (OR 4.97, 95% CI 3.31-7.52; *P*<.001), respectively, of being abstinent at 12 months. Descriptively, for QuitGuide, abstinence rates for the 2 trajectory groups were 21% for 1-week users and 23% for 3-week users. There was no significant difference in the odds of being abstinent at 12 months for 3-week versus 1-week users (OR 1.16, 95% CI 0.84-1.62; *P*=.37). The above models adjusted for the baseline covariates selected, as outlined in the statistical methods, and are shown in Table 1.

**Table 1:** Logistic regression models predicting 12-month smoking cessation outcome by log-in trajectory group, adjusted for Akaike Information Criterion model–selected covariates^a^.

Treatment arm and covariate			Odds ratio (95% CI)		*P* value
**iCanQuit**					
	4-week users	1.50 (1.05-2.14)		.03	
	26-week users	4.97 (3.31-7.52)		<.001	
	Gender (male)	1.87 (1.33-2.62)		<.001	
	High school or lower education	1.42 (1.03-1.95)		.03	
	Depression screen positive	0.69 (0.50-0.97)		.03	
	Panic disorder screen positive	0.74 (0.50-1.09)		.14	
	Used e-cigarettes at least once in past month	0.66 (0.45-0.96)		.03	
	Confidence in being smoke free	1.01 (1.01-1.02)		<.001	
	Drinks per day on a typical drinking day	0.95 (0.91-1.00)		.048	
**QuitGuide**					
	3-week users	1.16 (0.84-1.62)		.37	
	Used e-cigarettes at least once in past month	1.44 (0.99-2.08)		.05	
	Confidence in being smoke free	1.01 (1.00-1.02)		.005	
	Close friends who smoke	0.89 (0.81-0.97)		.01	
	Heavy drinker^b^	0.64 (0.37-1.07)		.10	
	Valuing questionnaire—progress	1.03 (1.01-1.06)		.01	

^a^The reference group is 1-week users for both arms.

^b^Heavy drinkers are defined as women who had 4 or more drinks and men who had 5 or more drinks on a typical drinking day.

### Baseline Characteristics Predicting Trajectory Membership

Because the trajectory groups were different across the 2 arms, Table 2 presents the results for baseline characteristics predicting membership in the groups for the 2 arms separately. For iCanQuit, the baseline characteristics significantly associated with more engaged group membership, as compared with 1-week user group membership, were age in years (OR 1.05, 95% CI 1.03-1.06 for 26-week users), smoking up to one-half pack per day (OR 1.90, 95% CI 1.25-2.87 for 26-week users), smoking first cigarette >5 minutes after waking (OR 1.42, 95% CI 1.05-1.92 for 4-week users), and higher mean acceptance of internal physical sensations (OR 1.82, 95% CI 1.41-2.35 for each 1-point increase for 4-week users).

For QuitGuide, the baseline characteristics significantly associated with 3-week user group membership, as compared with 1-week user group membership, were female gender (OR 1.46, 95% CI 1.10-1.95), minority race (people of color) or ethnicity (Hispanic; OR 1.40, 95% CI 1.08-1.83), and smoked for 10 or more years (OR 1.56, 95% CI 1.04-2.35).

**Table 2:** Multinomial logistic regression (iCanQuit arm) and logistic regression (QuitGuide arm) results predicting log-in trajectory group membership from Akaike Information Criterion model–selected baseline characteristics^a^.

Arm, trajectory group, and characteristic				Odds ratio (95% CI)
**iCanQuit**				
	**4-week users**			
		Age (years)	1.01 (0.99-1.02)	
		Smokes ≤10 cigarettes per day	1.25 (0.89-1.76)	
		First cigarette >5 minutes after waking	1.42 (1.05-1.92)	
		Number of quit attempts in previous year	1.03 (0.99-1.08)	
		Each point increase in acceptance of physical sensations	1.82 (1.41-2.35)	
	**26-week users**			
		Age (years)	1.05 (1.03-1.06)	
		Smokes ≤10 cigarettes per day	1.90 (1.25-2.87)	
		First cigarette >5 minutes after waking	1.42 (0.96-2.08)	
		Number of quit attempts in previous year	0.92 (0.83-1.02)	
		Each point increase in acceptance of physical sensations	1.23 (0.88-1.71)	
**QuitGuide: 3-week users**				
	Gender (female)			1.46 (1.10-1.95)
	Minority race or ethnicity			1.40 (1.08-1.83)
	Anxiety screen positive			0.75 (0.55-1.02)
	Smoked for ≥10 years			1.56 (1.04-2.35)

^a^The reference group is 1-week users for both treatment arms.

## Discussion

### Principal Findings

This study contributes to the nascent literature on longitudinal use trajectories of digital health interventions and their prediction of health outcomes [20-25]. The study found (1) 1-, 4-, and 26-week trajectories for iCanQuit versus 1- and 3-week trajectories for the QuitGuide smoking cessation apps; (2) that these trajectory groups differentially predicted smoking outcomes at 12 months for iCanQuit but not for QuitGuide; and (3) that certain user characteristics were associated with membership in certain trajectory groups. Notably, compared with the 1-week iCanQuit users, the 4-week users had 50% higher odds, whereas 26-week users had 397% higher odds, respectively, of being abstinent at 12 months. The results are discussed in the following sections.

### Use Trajectories and Health Outcomes

At least half of the participants in both arms were 1-week users. Similarly, our 2018 study examining log-in trajectories of 2 smoking cessation websites found that half of the participants were 1-week users, and similar to this study, that study showed that these participants were the least likely to have quit smoking at the 12-month follow-up [24]. Thus, there are now 4 separate digital interventions (2 in this study and 2 in the 2018 study [24]) showing large proportions of users (645/1309, 49.27% to 610/1069, 57.06%) who are 1-week users, suggesting overall that 1-week use may be a common engagement pattern of digital health interventions. Thus, it is imperative to identify early who would likely become a 1-week user. For example, the baseline characteristics results of this study suggest that a younger age, smoking at least one-half pack per day, smoking the first cigarette within 5 minutes of waking (a marker of nicotine dependence), and scoring lower on acceptance of internal physical sensations that trigger smoking (a marker of avoidance of cigarette cravings) predicted membership in the iCanQuit 1-week trajectory group. Measuring these factors at baseline might allow for the early identification of individuals who would be more likely to disengage from iCanQuit in the first week. Another approach that might be worth testing in future research is investigating use patterns within the first week (eg, number of log-ins per day and time spent on the app per day) to predict whether a participant would become a 1-week user. Once identified, more intensive intervention strategies could be used with this group, which might range from push notification communications or proactive intervention. Beyond this study, it would be worthwhile to determine whether 1-week use is a common pattern across multiple digital platforms (eg, websites and smartphone apps) and health domains (eg, tobacco, exercise, and diet) and to what extent this use pattern affects health outcomes.

The second trajectory group for each arm was the 4-week users for iCanQuit (303/1069, 28.34%) and 3-week users for QuitGuide (369/1064, 34.68%). Although the length of each group was similar (3-4 weeks) and the proportion of each group was somewhat smaller in iCanQuit, only the iCanQuit 4-week users had significantly higher quit rates than their 1-week comparators (ie, 50% higher odds of quitting). Two potential reasons why iCanQuit’s, but not QuitGuide’s, second trajectory group had higher quit rates are most likely due to the content and structure of the iCanQuit app. Regarding content, we have published multiple studies showing that the effect of iCanQuit (but not QuitGuide) on smoking cessation was mediated by ACT-based processes of acceptance of internal cues to smoke (ie, sensations, thoughts, and emotions) [35,45-48]. The differences in content, with iCanQuit focused on ACT versus QuitGuide focused on standard USCPG content [49], suggest that 4 weeks of engaging with ACT content that targets acceptance of internal cues is effective at improving quit rates. Regarding structure, the iCanQuit app presented content in a sequenced interactive format (eg, content is unlocked in a sequential manner) with short paragraphs of text and some audio or visual for experiencing ACT concepts, whereas the QuitGuide app presented content in a sequenced format with short paragraphs of text [35]. Thus, the extent to which intervention engagement predicts behavior change might depend on the content and structure of the intervention, which is a valuable topic for future research. For iCanQuit, these results suggest that strategies to increase engagement 3 more weeks (ie, from 1 to 4) could be an effective approach to improving quit rates. Example strategies worth testing include (1) proactive check-ins (via SMS text messages or phone calls) from staff about progress with the iCanQuit app, (2) rewarding each day’s use of iCanQuit, and (3) a “four-week challenge,” which shows other users’ daily log-in progress toward the goal of 4 weeks of use.

Only iCanQuit had a third trajectory group, namely 26-week users (156/1069, 14.59% of the iCanQuit arm sample). The 26-week users’ group had nearly 400% higher odds of quitting smoking (as compared with 1-week users). The 12-month 56% quit rates observed in this group are the highest we have ever observed in a digital smoking cessation intervention and suggest that iCanQuit could be a highly effective and scalable intervention for this group of users. Our 2018 paper found a similar group of long-term users on the WebQuit website (159/1240, 12.82% of WebQuit arm sample) who had high 12-month quit rates (34.2% [24]) but not as high as those found here for iCanQuit. The iCanQuit ACT-based content and structure may have encouraged long-term, spaced skills practice [6]. Taken together, the findings for both the iCanQuit and WebQuit third trajectory groups suggest that consistent use of each program over time is prognostic of a better health outcome, which is contrary to the notion that consistent log-ins may be a marker of ongoing challenges and struggles to change a health behavior. Instead, consistent log-ins over time may be a marker of a participant’s commitment to changing a health behavior. Digital intervention designs could focus on methods to encourage commitment and prevent lapses over time, which may include strategies similar to those suggested above, in addition to just-in-time adaptive interventions that aim to provide the right type of lapse-prevention support to smokers at the right time [50].

### Personal Characteristics and Use Trajectories

The impact of personal characteristics on use trajectories appears to vary according to the intervention. For example, among iCanQuit participants, smoking the first cigarette >5 minutes after waking and higher levels of acceptance of physical cues to smoke predicted being a 4-week user, whereas older age and smoking ≤10 cigarettes per day predicted being a 26-week user. The findings generally suggest that less dependence and greater acceptance of cravings predict long-term engagement with iCanQuit. The results on increasing age predicting iCanQuit’s 26-week use trajectory membership are consistent with past research showing that older age is a predictor of higher digital health intervention use [28-31], including our 2018 WebQuit trajectories paper [24]. In contrast to the view that as people age, their willingness to use technology decreases [51], this study suggests that increasing age may actually indicate which one is more likely to remain long-term users of iCanQuit, and in turn, have very high quit rates. In contrast, although there were baseline factors that predicted QuitGuide’s use trajectory membership, neither of the 2 trajectories predicted smoking cessation, so the value of these baseline prediction results is unclear. Nonetheless, we recommend that future research explore a variety of baseline subgroup differences (eg, sex, race, and age) in digital intervention trajectories to better understand who is most or least likely to engage over time. Overall, these analyses suggest a need for further research on which baseline factors might predict different use trajectories and therefore inform the development of tailored interventions that facilitate long-term, consistent engagement based on an individual’s specific baseline characteristics.

### Limitations

This study has several key limitations. First, only 2 smartphone apps were tested, and both were focused on smoking cessation; thus, future research should examine the extent to which the results generalize to other behaviors and to other types of digital interventions. Cessation outcome data were self-reported for the reasons stated in the Methods section. Remote biochemical validation of smoking cessation would have introduced biases including low response rates, prohibitive cost, challenges with confirming the identity of the person providing the sample, and inability to confirm abstinence beyond 24 hours [52-59]. Owing to a technical error, log-ins were recorded for the first 6 months of the trial. Finally, as users self-select to different app use patterns (rather than being randomized), the associations observed in this study may not be causal, and care should be taken in their interpretation.

### Conclusions

Patterns of 1-, 3-, and 4-week use of smartphone apps may be common for how people engage in digital health interventions. In addition, 4-week users, and especially 26-week users of iCanQuit, have higher odds of quitting smoking. Strategies to detect potential 1-week iCanQuit users and proactively offer them more intensive intervention could be fruitful.

## Appendix

Multimedia Appendix 1 Summary of baseline characteristics of participants from the iCanQuit and QuitGuide arms by log-in trajectories.

## Abbreviations

ACT: acceptance and commitment therapy
FPC: functional principal component
OR: odds ratio
PPA: point prevalence abstinence
USCPG: US Clinical Practice Guidelines
