# Supplementary material for: Smoking Cessation Smartphone App Use Over Time: Predicting 12-Month Cessation Outcomes in a 2-Arm Randomized Trial
Source: J Med Internet Res. 2022 Aug 18;24(8):e39208. doi: 10.2196/39208 (PMC9437788; doi:10.2196/39208)
Supplement: Multimedia Appendix 1 [file jmir_v24i8e39208_app1.docx]

**Multimedia Appendix 1.** Summary of baseline characteristics of participants from the iCanQuit and QuitGuide arms by log-in trajectories.

| Participant characteristics | | | | iCanQuit (n=1069) | | | | | QuitGuide (n=1064) | | | |
| --- | --- | --- | --- | --- | --- | --- | --- | --- | --- | --- | --- | --- |
|  | | | | Participants, n | 1-week users (n=610, 57.06%) | 4-week users (n=303, 28.34%) | 26-week users (n=156, 14.59%) | Overall (n=1069) | Participants, n | 1-week users (n=695, 65.32%) | 3-week users (n=369, 34.68%) | Overall (n=1064) |
| **Demographics** | | | | | | | | | | | | |
|  | Age (years), mean (SD) | | | 1069 | 36.7 (10.3) | 37.9 (11.0) | 42.2 (10.4) | 37.8 (10.7) | 1064 | 37.5 (10.8) | 38.4 (10.9) | 37.8 (10.9) |
|  | Sex (male), n (%) | | | 1069 | 186 (30.5) | 90 (29.7) | 42 (26.9) | 318 (29.75) | 1064 | 226 (32.5) | 90 (24.4) | 316 (29.70) |
|  | Race, n (%) | | | | | | | | | | | |
|  |  | | White | 1069 | 420 (68.9) | 224 (73.9) | 105 (67.31) | 749 (70.07) | 1064 | 494 (71.1) | 242 (65.6) | 736 (69.17) |
|  |  | | Black or African American | 1069 | 120 (19.7) | 49 (16.2) | 35 (22.4) | 204 (19.08) | 1064 | 125 (18.0) | 89 (24.1) | 214 (20.11) |
|  |  | | Asian | 1069 | 1 (0.2) | 3 (1.0) | 1 (0.6) | 5 (0.47) | 1064 | 1 (0.1) | 0 (0.0) | 1 (0.09) |
|  |  | | American Indian or Alaska Native | 1069 | 12 (2.0) | 3 (1.0) | 4 (2.6) | 19 (1.78) | 1064 | 16 (2.3) | 7 (1.9) | 23 (2.16) |
|  |  | | Native Hawaiian or Pacific Islander | 1069 | 1 (0.2) | 0 (0.0) | 1 (0.6) | 2 (0.19) | 1064 | 1 (0.1) | 1 (0.3) | 2 (0.19) |
|  |  | | Multiple races | 1069 | 44 (7.2) | 18 (5.9) | 10 (6.4) | 72 (6.74) | 1064 | 41 (5.9) | 28 (7.6) | 69 (6.48) |
|  |  | | Unknown race | 1069 | 12 (2.0) | 6 (2.0) | 0 (0.0) | 18 (1.68) | 1064 | 17 (2.4) | 2 (0.5) | 19 (1.79) |
|  |  | | Hispanic | 1069 | 64 (10.5) | 24 (7.9) | 10 (6.4) | 98 (9.17) | 1064 | 65 (9.4) | 31 (8.4) | 96 (9.02) |
|  | High school or less education, n (%) | | | 1069 | 261 (42.8) | 113 (37.3) | 60 (38.5) | 434 (40.60) | 1064 | 282 (40.6) | 147 (39.8) | 429 (40.32) |
|  | Working, n (%) | | | 1069 | 334 (54.8) | 181 (59.7) | 73 (46.8) | 588 (55.00) | 1064 | 392 (56.4) | 198 (53.7) | 590 (55.45) |
|  | Low income (<US $20,000 per year), n (%) | | | 1069 | 228 (37.4) | 111 (36.6) | 56 (35.9) | 395 (36.95) | 1064 | 236 (34.0) | 130 (35.2) | 366 (34.40) |
|  | Urban residence, n (%) | | | 1069 | 486 (79.7) | 242 (79.9) | 120 (76.9) | 848 (79.33) | 1064 | 528 (76.0) | 279 (75.6) | 807 (75.85) |
|  | Married, n (%) | | | 1069 | 183 (30.0) | 94 (31.0) | 47 (30.1) | 324 (30.31) | 1064 | 225 (32.4) | 116 (31.4) | 341 (32.05) |
|  | LGBT^a^, n (%) | | | 1069 | 110 (18.0) | 64 (21.1) | 27 (17.3) | 201 (18.80) | 1064 | 119 (17.1) | 67 (18.2) | 186 (17.48) |
| **Mental health, n (%)** | | | | | | | | | | | | |
|  | Positive depression screening results | | | 1064 | 606 (50.2) | 136 (45.0) | 71 (45.5) | 511 (48.03) | 1060 | 334 (48.3) | 178 (48.2) | 512 (48.30) |
|  | Positive anxiety screening results | | | 1063 | 167 (27.6) | 76 (25.2) | 27 (17.3) | 270 (25.40) | 1058 | 197 (28.5) | 83 (22.6) | 280 (26.47) |
|  | Positive panic screening results | | | 1052 | 168 (28.0) | 82 (27.4) | 39 (25.3) | 289 (27.47) | 1047 | 207 (30.3) | 98 (27.0) | 305 (29.13) |
|  | Positive PTSD^b^ screening results | | | 1057 | 287 (47.8) | 122 (40.5) | 53 (34.0) | 462 (43.71) | 1059 | 304 (44.0) | 157 (42.7) | 461 (43.53) |
| **Smoking behavior** | | | | | | | | | | | | |
|  | FTND^c^ score, mean (SD) | | | 1069 | 6.0 (2.0) | 5.6 (2.1) | 5.8 (2.0) | 5.9 (2.1) | 1064 | 5.9 (2.0) | 5.9 (2.0) | 5.9 (2.0) |
|  | High nicotine dependence (FTND≥6), n (%) | | | 1069 | 394 (64.6) | 166 (54.8) | 88 (56.4) | 648 (60.62) | 1064 | 411 (59.1) | 228 (61.8) | 639 (60.06) |
|  | Number of cigarettes per day, mean (SD) | | | 1069 | 19.4 (13.6) | 18.4 (13.8) | 17.5 (12.1) | 18.9 (13.4) | 1064 | 19.9 (15.5) | 18.8 (15.1) | 19.5 (15.4) |
|  | Smokes more than one-half pack per day, n (%) | | | 1069 | 470 (77.0) | 215 (71.0) | 103 (66.0) | 788 (73.71) | 1064 | 534 (76.8) | 268 (72.6) | 802 (75.38) |
|  | Smokes more than one pack per day, n (%) | | | 1069 | 127 (20.8) | 64 (21.1) | 29 (18.6) | 220 (20.58) | 1064 | 139 (20.0) | 70 (19.0) | 209 (19.64) |
|  | First cigarette within 5 minutes of waking, n (%) | | | 1069 | 357 (58.5) | 141 (46.5) | 75 (48.1) | 573 (53.60) | 1064 | 380 (54.7) | 197 (53.4) | 577 (54.23) |
|  | Smoked for ≥10 years, n (%) | | | 1069 | 485 (79.5) | 251 (82.8) | 144 (92.3) | 880 (82.32) | 1064 | 559 (80.4) | 320 (86.7) | 879 (82.61) |
|  | Used e-cigarettes at least once in past month, n (%) | | | 1069 | 147 (24.1) | 79 (26.1) | 38 (24.4) | 264 (24.70) | 1064 | 161 (23.2) | 87 (23.6) | 248 (23.31) |
|  | Quit attempts in past 12 months, mean (SD) | | | 1021 | 1.1 (2.4) | 1.9 (6.5) | 0.9 (1.6) | 1.3 (4.0) | 1009 | 1.4 (2.8) | 1.7 (10.7) | 1.5 (6.7) |
|  | Confidence in being smoke free, mean (SD) | | | 1069 | 63.6 (27.4) | 63.1 (28.1) | 67.5 (23.6) | 64.0 (27.1) | 1064 | 65.4 (26.6) | 63.8 (26.9) | 64.8 (26.7) |
|  | Friend and partner smoking | | | | | | | | | | | |
|  |  | Close friends who smoke, mean (SD) | | 1069 | 2.7 (1.7) | 2.6 (1.7) | 2.6 (1.7) | 2.7 (1.7) | 1064 | 2.6 (1.8) | 2.7 (1.7) | 2.6 (1.7) |
|  |  | Number of adults in home who smoke, mean (SD) | | 1069 | 1.5 (0.8) | 1.5 (0.9) | 1.3 (0.7) | 1.5 (0.8) | 1064 | 1.5 (0.9) | 1.5 (1.1) | 1.5 (0.9) |
|  |  | Living with partner who smokes, n (%) | | 1069 | 233 (38.2) | 104 (34.3) | 46 (29.5) | 383 (35.83) | 1064 | 246 (35.4) | 128 (34.7) | 374 (35.15) |
| **ACT^d^-based measure, mean (SD)** | | | | | | | | | | | | |
|  | Acceptance of internal cues to smoke | | | | | | | | | | | |
|  |  | | Sensations | 1058 | 3.0 (0.6) | 3.2 (0.6) | 3.1 (0.5) | 3.1 (0.6) | 1051 | 3.1 (0.6) | 3.1 (0.6) | 3.1 (0.6) |
|  |  | | Emotions | 1059 | 2.9 (0.5) | 2.9 (0.5) | 2.9 (0.4) | 2.9 (0.5) | 1060 | 2.9 (0.5) | 2.9 (0.5) | 2.9 (0.5) |
|  |  | | Thoughts | 1060 | 2.8 (0.5) | 2.9 (0.5) | 2.8 (0.4) | 2.8 (0.5) | 1062 | 2.9 (0.5) | 2.9 (0.4) | 2.9 (0.5) |
|  |  | | Mean score | 1054 | 2.9 (0.4) | 3.0 (0.4) | 2.9 (0.4) | 2.9 (0.4) | 1050 | 3.0 (0.4) | 3.0 (0.4) | 3.0 (0.4) |
|  | Valuing Questionnaire | | | | | | | | | | | |
|  |  | | Progress | 1057 | 18.8 (7.7) | 19.0 (7.4) | 19.7 (7.4) | 19.0 (7.6) | 1053 | 19.4 (7.5) | 19.7 (7.5) | 19.5 (7.5) |
|  |  | | Obstruction | 1056 | 12.1 (8.6) | 11.2 (8.3) | 10.7 (8.4) | 11.7 (8.5) | 1056 | 11.2 (8.3) | 11.5 (8.4) | 11.3 (8.4) |
| **Alcohol use** | | | | | | | | | | | | |
|  | Drinks per day on typical drinking day, mean (SD) | | | 1038 | 2.0 (4.1) | 1.9 (3.6) | 2.2 (4.8) | 2.0 (4.1) | 1033 | 1.9 (4.0) | 1.5 (2.6) | 1.8 (3.6) |
|  | Heavy drinker^e^, n (%) | | | 1038 | 87 (14.7) | 47 (16.0) | 27 (17.5) | 161 (15.51) | 1033 | 99 (14.7) | 47 (13.1) | 146 (14.13) |

^a^LGBT: lesbian, gay, bisexual, and transgender.

^b^PTSD: posttraumatic stress disorder.

^c^FTND: Fagerström Test for Nicotine Dependence.

^d^ACT: acceptance and commitment therapy.

^e^Heavy drinkers are defined as women who consumed 4 or more drinks and men who consumed 5 or more drinks on a typical drinking day.
